# Supplementary material for: Identification of photoperiod-induced specific miRNAs in the adrenal glands of Sunite sheep (Ovis aries)
Source: Front Vet Sci. 2022 Jul 22;9:888207. doi: 10.3389/fvets.2022.888207 (PMC9354845; doi:10.3389/fvets.2022.888207)
Supplement: Supplementary file 1 [file Data_Sheet_1.ZIP › Supplementary file/miRDeep2 file.zip!/miRDeep2 file/Novel_Structure/LP42A1/Novel_642.pdf]

Provisional ID	:	NC_019460.2_8679
Score total	:	46.6
Score for star read(s)	:	3.9
Score for read counts	:	43.5
Score for mfe	:	-2.3
Score for randfold	:	1.6
Score for cons. seed	:	
Total read count	:	97
Mature read count	:	83
Loop read count	:	0
Star read count	:	14

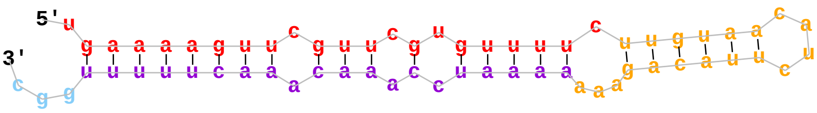

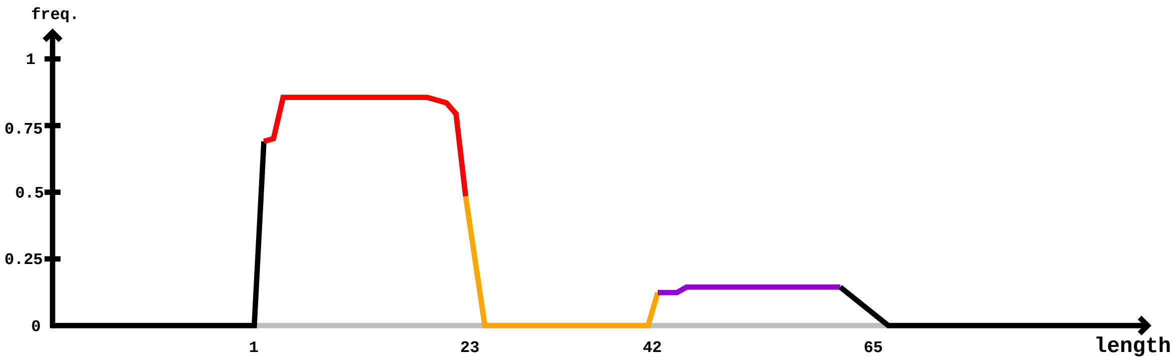

Mature Star

```
5'- cuguaaaggcaacagagggcugaaaaguucguucguguuuucuuguaacaucuuacagaaaaaaauccaaacaaacuuuuuggccaacccaguauauucaacaggacucuug   -3'    obs
    cuguaaaggcaacagagggcugaaaaguucguucguguuuucuuguaacaucuuacagaaaaaaauccaaacaaacuuuuuggccaacccaguauauucaacaggacucuug           exp
    ((((.......))))..((((((((((((.(((.(.(((((.((((((....))))))...))))).).))).))))))))))))...((.((.......)).)).......    reads     mm      sample
    ....................ugaaaaguucguucgugu..........................................................................    1         0       seq
    ....................ugaaaaguucguucguguu.........................................................................    1         0       seq
    ....................ugaaaaguucguucguguuu........................................................................    4         0       seq
    ....................ugaaaaguucguucguguuuu.......................................................................    29        0       seq
    ....................ugaaaaguucguucguguuuuc......................................................................    32        0       seq
    .....................gaaaaguucguucguguuuu.......................................................................    1         0       seq
    ......................aaaaguucguucgGguuuuc......................................................................    15        1       seq
    .............................................................aaaauccGaacaaacuuuuu...............................    6         1       seq
    .............................................................aaaauccaaacGaacuuuuu...............................    3         1       seq
    .............................................................aaaauccaaacGaacuuuuug..............................    1         1       seq
    .............................................................aaaauccaaacGaacuuuuugg.............................    2         1       seq
    ................................................................auccGaacaaacuuuuuggcc...........................    2         1       seq
```
